# American Academy of Dental Science

**Published:** 1902-05

**Authors:** 

**Affiliations:** American Academy of Dental Science


### AMERICAN ACADEMY OF DENTAL SCIENCE.

The regular monthly meeting of the American Academy of Dental Science was held at Young's Hotel, Boston, on Wednesday evening, January 9, 1902, President Bradley in the chair.

Mr. George B. Gordon, of the Peabody Museum of American Archæology and Ethnology, Harvard University, was the principal guest and essayist of the evening.

The Academy first gave its attention to incidents of office practice and presentation of specimens.

*President Bradley.*—We will take this occasion for presentation of instruments and specimens. If there is anything that the fellows have to offer, we shall be pleased to give them this opportunity to present it to the Academy.

*Dr. Delabarre.*—I have a Logan crown which was worn in the mouth perhaps a year, or a little over, when the pin broke. The patient came to me and told me that she was not conscious of any strain which she put upon the tooth at all, but she realized the crown was off one day, and found it on her tongue. I looked at it closely, and to the naked eye it was evident that the platinum in the pin was crystallized. Never having heard of anything of the kind before, I thought I would present it to the Academy. You will see the pin is crystallized; through a microscope it shows up much better. Opposite that is a platinum pin which I took this afternoon for the sake of comparison, and with two pairs of pliers it took ten full bends one side and the other before it broke; and you can see that pin is not crystallized like the other is.

*Dr. Baker.*—I would like to ask Dr. Delabarre how that pin was fastened in?

*Dr. Delabarre.*—With cement. I know that steel will crystallize under vibration in machinery and bicycles, so that under very slight strain it will break. I have had handle-bars on my bicycle break easily. It comes off and is perfectly crystallized, the same as zinc ore. I never knew platinum to do that, and particularly a case where it is so firmly surrounded by cement and there can be no vibration.

*President Bradley.*—As I look at the specimen, it seems to me, Dr. Delabarre, that the crystallized appearance is possibly the burnishing. That is what I should have supposed it was, unless you had told me it was crystallization. It seems to me as if it had been burnished against the broken part of the tube and got a little polish on it at certain points.

*Dr. Delabarre.*—I think I should differ with you on this ground, —that if it were polished by burnishing, it would not have the sharp and fine angles that that has.

*Dr. Stevens.*—I should like to ask Dr. Delabarre if he knows what per cent. of platinum is in these pins.

*Dr. Delabarre.*—I do not. I did not put it in myself, and do not know, and have no means of ascertaining how much platinum there is in it.

*Dr. Banfield.*—Do I understand this pin was broken, or came out of the tooth only?

*Dr. Delabarre.*—The pin was badly broken. I had to spend about two hours on it.

*Dr. Banfield.*—Why I asked is this: I had occasion to have a tooth baked a few days ago, and I had a platinum pin baked in it. I afterwards tried to bend the pin and found that it broke very easily; it seemed brittle, where before the metal seemed hard and tough. This changed condition in the platnium might be explained if overheated. I have never had this happen before with any platinum pin.

*Dr. Meriam.*—What furnace was used for baking?

*Dr. Banfield.*—A gas furnace.

*Dr. Werner.*—I had the same experience that Dr. Banfield speaks of. Many of the crowns baked on platinum bases are decidedly unsatisfactory for that reason. The excessive heat used in baking them often causes failure, not only from the brittleness of the pins and platinum base, but from the porcelain shrinking away. If you have it thin and burnish it down to fit the root, you will have to have it re-enforced, sometimes the third time, before you have a satisfactory baking on the floor. Is not pure platinum made brittle by heating or hammering it out? Is the metal deteriorated by flattening or heating? Does a molecular change take place? Platinum, somehow or other, seems very unsatisfactory, surprisingly so at times.

*President Bradley.*—The paper for this evening, entitled "Among the Honduranians, Ancient and Modern," is something that we may look forward to with anticipations of a great deal of pleasure. The country is one of great interest to us, being in Central America, and we shall undoubtedly listen to the address with much interest. Mr. Gordon has spent some time in that region, travelling and making expeditions and explorations. I have very great pleasure in introducing to you Mr. George Byron Gordon, of the Peabody Museum of Harvard University.

(For Mr. Gordon's paper, see page 321.)

DISCUSSION.

*President Bradley.*—I am sure we have all been interested in this story of the Honduranians, and shall be pleased to continue the discussion, which is to be opened by Dr. Fillebrown.

*Dr. Fillebrown.*—Mr. President, I hope to say a few words, and at this time, I think, if our guest will give us the benefit of his photographs, by passing them around, it will add to the interest of the discussion.

*Mr. Gordon.*—This first photograph is not from Copan, but from the ruins of Panama, in Yucatan. It shows a complete skull, the skull of a young girl, apparently, which shows the filing of the teeth. It is a quite perfect specimen.

The second shows a skull from somewhere on the peninsula of Yucatan, the exact locality being unknown. The jewels which had been set in the front teeth have all fallen out, but you still see the cavities in which they were laid.

The third photograph represents several sets of teeth from the tombs of Copan.

*Dr. Fillebrown.*—These remains, I understand, date somewhere from one to two thousand years ago?

*Mr. Gordon.*—About a thousand years. The limit in the other direction might well be placed at two thousand.

*Dr. Fillebrown.*—This photograph is particularly interesting because it comes right home to us, and especially to Dr. Allen, who gave us a paper on inlays at our last meeting. It shows remarkably well the perfection of the circular inlays, and it is surprising to what a degree of perfection they attained.

I had the privilege, not long since, of seeing the originals from which these photographs were taken, and it was hoped that Mr. Gordon would be able to have them here this evening; but they are so specially rare and so very valuable that the superintendent of the museum was unwilling that they should go out of the custody of the museum, even for a short time, and consequently we do not have the privilege of seeing them. But any one who can find the time to call at the museum can examine them.

What I have to say is of the dental aspect of the case. These inlays were made of what is called green jade, and are very accurately fitted. They were put in for the purpose of ornament, undoubtedly, and for nothing else. Several of them I saw there that were perfect in position and have remained so through all the disintegrating power of the years that have intervened. Some of them were out, and the cavities were still perfect. In others decay had destroyed the formation of the cavity. But it is remarkable that they could be so perfectly fitted. There is no man among us to-day that can cut a cavity and put in an inlay more perfectly than those are put in. They were supported by a cement, white in some cases, in other cases red. In some of the cavities there was a red substance, which seemed to be of the nature of cement. These were brought to Cambridge in 1893, or a little before that. The discoverer of them and many of the authorities of the museum consider that they belong to one set. It was reported that they were found in place in the jaw; but I think that cannot be so, for so important a part of a specimen would not have been left behind. Among these you will notice one which represents rudely the root of a tooth.

In a communication to the *Dental Practitioner,* then published in Buffalo, these specimens are referred to and the opinions of the collector and some other observers are stated. The statements I wish to speak of are in regard to the supposed artificial root carved from stone which appears in the case with the tooth. The first statement is that it was found neatly fitted in the socket of an inferior left lateral incisor, and was shaped very much like a natural tooth. It is also stated that these all belong to one set of teeth. To my mind they are not all of one set, and I think any careful observer would say that the teeth were disproportionate; and it is questionable whether on one side one bicuspid is not represented by a canine. But I am satisfied in my own mind that they are not all from one set of teeth.

The second statement is that it had been worn some time during life, and this was indicated by a thick incrustation of tartar on it. I understand that this opinion was held by men of prominence for erudition in archæological knowledge who are quoted as authorities on these subjects. After closely examining the specimens lately, the opinion seemed to be forced upon me that the accuracy of these opinions is questionable, and I am obliged to differ from them for the following reasons:

1. It seems to me highly improbable that, had the root and teeth been found in the jaw, the discoverer would have failed to preserve them in place, and the jaw would be with the teeth. Indeed, no whole bones were found, only the crumbled fragments left by the disintegrating force of time.

2. These teeth which are held on a card of wax are, to my mind, evidently not all of the same set. If so, this shows that the remains were somewhat mixed, in which case foreign matters might have been picked up with them.

3. Any artist, capable of carving the root of a tooth to fit the socket in the jaw, would not have stopped there, but would have finished the crown also. The probability of its being broken after it was made is very remote.

4. It is proved that nature will not tolerate any substance so rough and generally incompatible as is this alleged root.

5. Careful recent examination of this alleged root has proved it to be the point of a stalagmite, and this alleged tartar the partially crystallized lime salts which make up the additions to the main body.

I think it well that these statements go on record somewhere, because it seems to me that the prior statements that have been made in regard to it, and I have good reason to believe are entertained by credible men at the present time, should not pass too far down into history without the counter-statements regarding such apparently incorrect conclusions.

I cannot presume to add a word to the most excellent and interesting story that our friend has given us to-night. I for one thank him for being willing to come here and tell us of it. There is much of interest added to any account when the man is talking who has lived there himself, in regard to what he has done and what he has seen. It gives a life-like vividness to it that no written account can awaken.

*President Bradley.*—The subject is open for general discussion. We should be pleased to have the matter referred to in any way, or questions asked. I have no doubt Mr. Gordon would be glad to answer any questions.

*Dr. Stoddard.*—I would like to ask Mr. Gordon if there is any point of similitude in the things that are found in these ruins to those of the mound builders,—*i.e.,* any of the instruments, or pottery, or anything of that sort, that are found in the mounds in the Western and Southern States?

*Mr. Gordon.*—So far as the pottery is concerned, there are certain types of pottery that are common to all localities in America, and affinities to these types could be found in almost any part of the world. Outside of these types, that might be called common types and might pertain to almost any country in the world, I have noticed no affinities. The decoration on the pottery found in these old cities appeared to me to be entirely distinct from any decoration that has been found anywhere in North America; the motives seem different, the designs entirely distinct, quite unique, and peculiar to themselves.

With regard to other things that have been found, it has been thought by some that in some of the ornaments and engravings on bone, shell, and stone, that have been dug up in the mounds in the valley of the Mississippi and its tributaries, have been found the rudimentary elements from which some of the motives in the symbolism of Central America can be traced; but there are great differences of opinion on this point, and some maintain that where similarities and apparent affinities come in it is to be regarded as purely accidental, and that it can be explained in that way.

*Dr. Clapp.*—What was the character of the stone and the depth of the excavations?

*Mr. Gordon.*—With regard to the depth of the excavations, it varied. When we undertook the excavation of any particular group of structures our plan of operation was, in the first place, to make a careful survey of the structure as we found it. It would present the appearance of a mound in the shape of a beehive, and all over it could be seen fragments of sculpture and of square building-stone. By excavating towards the centre of such a mound at the base for a distance varying from ten to twenty-five feet, we would come to the foundations of the building, with the lower walls still standing to the average height of five feet. All this mass of material removed by excavation was simply from the ruins of the upper part of the structure, which had fallen down and buried the lower parts of the structure in its own ruins. Sometimes enormous quantities of material consisting of broken sculptures, building-stone, and stucco had to be removed before the interiors of the buildings could be reached, which proved that these buildings were not only very broad in their foundations, but apparently must have extended to a great height.

The most important tombs were found, as a rule, beneath the pavements of court-yards, and for that reason it was very difficult to find them. You might remove a whole pavement and find no tomb at all; and then come upon one accidentally when least expecting it. There was nothing whatever to mark the position of these tombs. Each tomb was a simple vault, averaging about eight feet long and four feet wide, built of neatly cut stone, regularly laid in rows, without mortar or cement, the same kind of construction that was used in all the buildings. There was usually not more than two feet between the top of the pavement and the roof of the tomb. In other cases the tombs were found beneath the foundations of the buildings. All the buildings rest on pyramidal foundations, and the approach was by a flight of stone steps, leading up the slope to the entrance. Very frequently by removing a few of these steps and digging down to the level, or slightly lower than the level of the surrounding pavement, you would find a tomb. But not all the houses we explored had tombs connected with them in this way. These would seem to have been family vaults, and all of them contained the remains of more than one person.

The kind of stone that was used in all the structures, in the monoliths, walls of the temples, and everything, in fact, was a trachyte, a rock of volcanic origin, a species of tufa, not very hard. It is fine grained, and slightly softer than sandstone. The stones were all very nicely cut and squared, and mortar was not used in the construction. The builders were not careful to break joints, and that was one weakness in all the structures which facilitated their gradual disintegration.

*Dr. Werner.*—I would like to ask Mr. Gordon if, where these burial-places were, human bodies exclusively were buried, or whether there were remains of animals, or of teeth other than human teeth.

*Mr. Gordon.*—It cannot be said that there is any positive proof of a general custom of placing animal remains with the dead, although there was some evidence that would seem to point to such a custom. We did not find any teeth but human teeth in the tombs except in one instance. In this instance of which I speak the tomb had already been opened: one of the slabs forming the cover had been removed, and it had in course of time become filled up with débris and earth that had fallen in. Among this intrusive material near the bottom, and appearing to be in contact with the human remains, was found the tooth of a horse. It has been suggested that this discovery might have a bearing on the question of a native horse in America, but from the fact of the tomb having been opened, the evidence is worthless. The probability is that it had been introduced in recent times.

I should mention one case recorded by Maudslay. No vault had been built in this case, but a mound had been constructed about five feet high, and with a human skeleton in the interior of this mound was found the skull of a jaguar, which was painted red. It certainly was placed with the body at the time of burial, perhaps in fulfilment of some superstitious rite. In one case we found the skull of a peccary, or wild hog, which was beautifully carved over the entire surface. In another case there were several small jars found, and in these jars were the skeletons of small birds. These were the only instances in which remains other than human remains were found.

*Dr. Werner.*—Were any metals found?

*Mr. Gordon.*—No, no metals of any sort. That was one of the surprising things. Any one who looked at the sculptures would conclude that metal tools must have been used in making them. Sometimes the carving was very deep, and a great deal of under-cutting. It is difficult to conceive of a stone tool being formed long enough and at the same time strong enough and sharp enough to do that work; but no tools except stone tools were found, and no metals, not even gold. It is the rule for the streams in that country to contain gold washings, and yet the people who built Copan either did not make the discovery, or else they did not have any appreciation of the yellow metal.

*Dr. Clapp.*—Any specimens of glass found?

*Mr. Gordon.*—None except natural glass, that is, volcanic glass or obsidian. This material was used for making knives and implements requiring a very sharp edge. The knives were formed by flaking. A cylindrical block was formed, and then by some process, which we do not know, flake was broken off from one end to the other, giving a double-edged knife with very fine edges and a very fine point. The same material was used for making knives in Mexico at the time of the Conquest, and we find it stated in the letters written by the conquerors, that in the market-place of Mexico there were barbers' booths where Montezuma's warriors were shaved of their scanty beards with razors made of obsidian.

*Dr. Wilson.*—I would move that a vote of thanks be extended to Mr. Gordon for his exceedingly interesting and instructive talk this evening.

Voted.

Upon motion, adjourned.

CHARLES H. TAFT, D.M.D.,
*Editor American Academy of Dental Science.*

